# Governor Hughes’s Influence in the Tuberculosis Crusade

**Published:** 1910-11

**Authors:** 


### Governor Hughes's Influence in the Tuberculosis Crusade.

WHEN representatives of the State Charities Aid Association waited upon Governor Hughes just before he left Albany to assume his new duties at Washington as associate justice of the Supreme Court, to express their appreciation of his many services in the tuberculosis campaign in this state, the Governor said in substance that no matter how men differed as to political theories of government they should unite and should work in harmony to drive tuberculosis out of existence. These words recall with striking force that only recently the conventions of the two great political parties in this state, adopted platforms recognising the campaign against tuberculosis as demanding serious legislative consideration.

Such a statement from Governor Hughes comes with eminent authority, for it was he who fired one of the first and most powerful guns in the campaign against the Great White Plague in this state. At the first meeting of the crusaders in Albany, January, 1908, he said:

> If we had through the misfortune of war, or the sudden rise of pestilence, or through some awful calamity, the destruction of life that annually takes place on account of the spread of tuberculosis, we should be appalled, and mass meetings would be held in every community and demand would be made that the most urgent measures should be adopted. It is only because we are accustomed to this waste of life and are prone to think that it is one of the dispensations of Providence that we go on about our business, little thinking of the preventive measures that are possible.

This shot has truly been heard around the world, for the statement has been quoted in practically every civilised country to call public attention to the urgency of the tuberculosis problem.

And Governor Hughes has backed up his words with effective deeds. He signed the county hospital bill, which has already resulted in the establishment of or appropriations for ten county tuberculosis hospital sanatoria. The boards of supervisors in fifteen other counties also are investigating the subject. Ten city tuberculosis hospitals have either been established or appropriated for, and most of them have required special legislation which Governor Hughes has signed. The antituberculosis campaign in this state owes to Governor Hughes, also, a large measure of the public enthusiasm which has resulted in the establishment of the nine camps, the sixteen dispensaries, the thirty-five visiting nurses, and other local relief measures which are going a long way toward making good the slogan of the crusade, "No Uncared-for Tuberculosis in New York State in 1915."

